# Supplementary material for: BIPS—A code base for designing and coding of a Phage ImmunoPrecipitation Oligo Library
Source: PLoS Comput Biol. 2022 Nov 10;18(11):e1010663. doi: 10.1371/journal.pcbi.1010663 (PMC9681064; doi:10.1371/journal.pcbi.1010663)
Supplement: S1 Text — (DOCX) [file pcbi.1010663.s001.docx]

# **Supplementary Materials**

## S1 Text: Test Data

We exported all epitopes with IRIs (Internationalized Resource Identifiers), from IEDB [1] (<https://www.iedb.org/>, as of March 6, 2022). We downloaded to sets of epitopes both from a human host, B cell assay, linear epitope peptides of Infectious diseases and of Allergic diseases. The downloaded files contained 77,345 and 5,267 epitopes respectively.

For each of the two disease types, we filtered only epitopes for which the parent protein (column ‘Epitope/Parent Protein IRI’ of the downloaded epitope file) originated from uniprot [2]. After considering only unique parent proteins, we downloaded the full protein sequences from the uniprot api, and created a protein file.

References

[1. Vita R, Mahajan S, Overton JA, Dhanda SK, Martini S, Cantrell JR, et al. The Immune Epitope Database (IEDB): 2018 update. Nucleic Acids Res. 2019;47: D339–D343. doi:10.1093/nar/gky1006](https://sciwheel.com/work/bibliography/6079894)

[2. UniProt Consortium. UniProt: the universal protein knowledgebase in 2021. Nucleic Acids Res. 2021;49: D480–D489. doi:10.1093/nar/gkaa1100](https://sciwheel.com/work/bibliography/10304708)
