# Supplementary material for: BIPS—A code base for designing and coding of a Phage ImmunoPrecipitation Oligo Library
Source: PLoS Comput Biol. 2022 Nov 10;18(11):e1010663. doi: 10.1371/journal.pcbi.1010663 (PMC9681064; doi:10.1371/journal.pcbi.1010663)
Supplement: S2 Text — (DOCX) [file pcbi.1010663.s002.docx]

# **Supplementary Materials**

## S2 Text: Misidentification at 3’ End

If the barcode of an oligo, either internal or external, is at the 3’ end of an oligo then (BIPS by default uses the 5’ configuration) it is not enough to read the barcode from the end of the oligo. If a phage got multiple inserts of oligos in the cloning process (more than one oligo cloned into a single phage, by oligonucleotide restriction sites ligating to one another and creating 3, 5 or more oligonucleotide sequences in the same phage, the first in the original direction the second in reverse etc.), the peptide produced on its surface would be of the first oligo inserted, while the barcode read would be of the last oligo inserted, Thus, a short read from the start of the oligo is needed in order to ensure a match of the peptide actually produces, with the one identified by the barcode.

If barcoding is done at the 5’ end, something that cannot be created with external barcoding, then no such problem arises.
