# Supplementary material for: BIPS—A code base for designing and coding of a Phage ImmunoPrecipitation Oligo Library
Source: PLoS Comput Biol. 2022 Nov 10;18(11):e1010663. doi: 10.1371/journal.pcbi.1010663 (PMC9681064; doi:10.1371/journal.pcbi.1010663)
Supplement: S3 Text — (DOCX) [file pcbi.1010663.s003.docx]

# **Supplementary Materials**

## S3 Text: Post - Sequencing Usage

The following are steps that should be taken after sequencing samples with the library as part of the PhIP-Seq complete pipeline. Examples of this code appear in the GitHub repository, however, we suggest users implement the code themselves for their own use cases.

## Finding the Source of a Barcode

By design every sequenced barcode, with up to *n* errors (either read or synthesis errors) can be uniquely identified, but these barcodes were not designed to be able to deal with insertions or deletions. However, in most cases the barcodes are able to deal with 1-2 indels (and up to *n-1* or *n-2* errors), and still identify the source of the oligo. This stems from the differences in distributions of different shifts in a protein-derived oligonucleotide sequence. Of note, there is no way to tell if the indel is a read-error or a synthesis error, thus the true amino-acid sequence of the bound peptide is not known. As a synthesis error may be translated to a deleterious change to the peptide sequence, BIPS allows identification with or without allowing for indels for that purpose, and we suggest users identify their needs accordingly.

## Oligo Source

The output library spans the entire input protein set, where each protein in the original list is fully covered with overlapping oligos. If the same peptide is derived from two source proteins, it does not get coded twice, but the final oligo is marked with all the original protein and locations it was derived from. Furthermore, each final oligo is mapped to all proteins containing it (e.g if the same peptide as was split from one protein, appears in another protein but not at a position at which the second protein was split). These two lists of sources allow for the analysis of the bound peptides, on the one hand maintaining a set of oligos which uniformly covers each input protein, while on the other considering all possible sources of a bound oligo.
