# Supplementary material for: BIPS—A code base for designing and coding of a Phage ImmunoPrecipitation Oligo Library
Source: PLoS Comput Biol. 2022 Nov 10;18(11):e1010663. doi: 10.1371/journal.pcbi.1010663 (PMC9681064; doi:10.1371/journal.pcbi.1010663)
Supplement: Attachment — Submitted filename: Point_by_point.docx [file pcbi.1010663.s004.docx]

We thank the reviewers for the useful comments. We have revised our manuscript and addressed the comments raised by the reviewers and editor, and we believe that the manuscript is now substantially improved. Below is a point-by-point response to reviewers’ comments, and a new manuscript, with changes highlighted, is attached.

After having re-submitted this manuscript, with corrections described below, we were asked by the editor to reformat the paper in the form of a Software article. This reformatting moved significant parts of the manuscript around, and makes tracking changes very difficult. In order to make it easier to follow we included the revised manuscript (with and without track changes) before the re-formatting, along with the final re-formatted paper.

Reviewer #1: BIPS is a comprehensive tool to generate a Phage Immuno-precipitation library from Protein/peptide sequences of interest. The tool generates a library with desired oligo length and overlap.

Novelty of the code lies in the embedded barcode. In commonly used methods the barcode is at the 5p end of the sequences that reduces the effective length of the oligo or on the 3p end that makes paired end sequencing necessary.

This tool also ensures appropriate hamming distance between oligos.

This tool generates a barcode of desired length within the sequence with an elegant use of a codon optimization table. Internal barcoding reduces sequencing length requirements.

I have some minor comments:

1. Does the codon optimization reduce the probability of expression of that oligo, if there are large number of similar sequences?
In the case where there are multiple codons of the same sequence, this is possible, however, in the analysis of the entire PhIP-Seq this is taken into account and does not cause a difference between oligos, as we have shown in *Vogl et al. 2021.*
We’ve added the following text:
“It is clear that coding many identical (or nearly identical) sequences in a single library will increase the use of relatively rare codons, however we have shown that this does not cause a significant reduction in differential expression. “

2. Would it be possible to use the code without using internal barcoding strategy?
This is not implemented as part of our library as it seems like an unlikely use on our part. However, if one wishes to add an external use it is possible to define a single barcode with the length of the constant length peptide in base-pairs (e.g., 64aa*3 in the common case). Since all peptides are unique this will not affect the coding. At this stage one could either sequence the entire oligonucleotide or add an external barcode.

Reviewer #2: Leviatan et al have developed an informatics pipeline for converting protein sequences into DNA sequences encoding peptides to tile across the desired proteins (BIPS). The DNA sequences are then to be cloned en masse into phage display for the PhIP-Seq assay. PhIP-Seq has emerged as a powerful antibody profiling technology, so open source tools for library design will certainly be welcomed by users – especially since there remains a lack of established computational tools and standards. However, there are several unfortunate shortcomings of this manuscript that must be addressed before it is suitable for publication.

Major Issues

• Perhaps the most critical problem with this paper is that the open-source pipeline currently in use for PhIP-Seq library design, pepsyn (Mohan et al. Nat Prot), is not even referenced. Since it is not referenced, BIPS cannot be directly compared to the de facto standard.
This was a crucial reference, we were aware of the work but had not dove into the pepsyn software in the past.
“To this end, shorter input proteins or peptides are terminated by a stop codon, and padded with a random amino-acid sequence, as implemented in Pepsyn (M*ohan et al. 2018)*.”

• A second critical issue is that codon-based barcoding has been incorporated into the design of multiple previously published PhIP-Seq libraries. It has been utilized in at least two contexts of which I am aware. In the first, Xu et al (Science) used codon barcoding to distinguish alanine scanning variants of public epitopes. In the second example, Shrock et al (Science) used codon barcoding to distinguish duplicate peptides that enhance assay robustness. Xu is referenced but not in the context of codon barcoding, and Shrock is not referenced at all. Since these prior examples are not referenced, the approach used by BIPS cannot be compared or contrasted.
We appreciate the notes and have re-read the papers to compare and contrast.
“BIPS is intended for large peptide libraries and through the use of synonymous mutations, which was previously suggested (Xu et al. *2015) (Shrock et al. 2020)*, enables…”

“The concept of utilizing synonymous codons to allow unambiguous mapping has been previously discussed (Xu et al. *2015) (Shrock et al. 2020)*, here, we have advanced this approach by using it in order to ensure a hamming distance between sequences. ”

• The overall rationale for embedding codon barcodes is not articulated. It seemed to me that the main reason the authors would use codon-barcoding is for disambiguation of similar library members. However, for typical libraries and typical read lengths (say 50 nucleotides at minimum, but typically longer reads at incremental additional cost), the number of ambiguous library members would be extremely small. Of course, any number of ambiguous sequences is undesirable, but rescuing these sequences might not be the most compelling rationale. I suggest the authors emphasize additional reasons why codon barcoding is useful. Mutation scanning, deep mutagenesis, and peptide duplication are what I think of as the most important examples, but there are likely others. Reducing read length requirements might also be compelling to some users, but more specifics should be provided on the actual benefit for standard libraries and standard read lengths.
We were definitely focused on our own cases and did not consider cases where there is no benefit to barcoding peptides. We have added a motivation to embedded barcodes, and to barcoding in general:
“In cases of libraries where there are many similar peptides due to research of deep mutagenesis, mutation scanning, homologous proteins or other, partially sequencing a peptide might not be sufficient for identification, and alternative methods must be explored.”

In addition, the usage of our barcoding promises the ability to withstand sequencing errors on top of identification, this due to the predefined hamming distance between all pairs of barcodes.

• The authors are inconsistent with the PhIP-Seq abbreviation. PhageIP-Seq is used occasionally, but it is unclear if this is intentional or in error. The most commonly used long form seems to be Phage ImmunoPrecipitation Sequencing (with no hyphen like what is in the title).
Thanks for noticing! We fixed all such inconsistencies.

Minor Issues

• In the first paragraph, it is mentioned that peptide arrays have at most a few thousand antigens. There are certainly examples of more than a few thousand antigen arrays.

We stand corrected. ELISAs are usually limited to a few hundreds or thousands of oligos, but other peptide arrays can indeed get to tens of thousands. Phrasing changed to:

”in contrast to peptide arrays and enzyme-linked immunosorbent assays (ELISAs) which have limited capacities, of at most a few thousand or a few tens of thousands of antigens.”

• Padding shorter peptides with random amino-acid sequences downstream of a stop codon is also a feature highlighted in the pepsyn software so should be referenced as such.
Added the much needed reference.

• Typo “DNWA”
Changed.

• It is mentioned that the 3’ suffix must start with a stop codon. However, many users may want to have an epitope tag or some other fusion peptide C-terminal to the peptide. Requiring a stop codon would also make the design incompatible with M13 phage display, which may also be of interest to some of your readers.
We have made the modification both in our paper and in our repositories config file:
“In order to produce the protein, a stop codon must appear downstream of the peptide, it is suggested to include this within the suffix, (at 3’ end), but it is not mandatory (in case C-terminal tags should be added or M13 phage display should be used).”

• It is not clear how restriction sites are avoided during the peptide coding process. Could you provide additional information?
This was actually better explained in the repository and we have added it to the paper. In essence there are two iterations to see if a restricted sequence is within the coding, each resulting in a re-coding. The first is upon transforming the proteins into nucleotide sequences, the second is upon barcoding.
“All peptides must be coded using the host bacteria’s codon usage table, without including restricted sequences.”
“If a constructed barcode does not adhere to the hamming-distance or the entire peptide includes restricted sequences, BIPS re-codes the barcode until either getting a successful barcode, or until reaching a pre-defined number of iterations.”

• I had a hard time evaluating Fig 2b/c since the font was so small.
We have increased the font, but also added an SVG file for your comfort.

• I believe the Y-axis title of Fig 2b has a typo.
Fixed.

• The term “infectious diseases” is used where I think it is meant “Infectious organisms”.
This is the term that appears in the IEDB database, we have changed all occurrences to “IEDB’s infectious diseases”.

• In the paragraph above “Code Availability” section, FASTA files are referred to, but I believe it is meant “FASTQ”.
Fixed.

• Define “IRIs”.
Added the definition.

• At the end of the Test Case section, the number 24,456 appears to have been incorrectly copied from above. The replacement should be 1,745 if I am not mistaken.
Thanks for noticing! We corrected the error.

• In the “Finding the Source of a Barcode” section, the authors suggest doing sequencing read mapping by alignment with errors allowed. However, certain oligo synthesis errors may prematurely truncate the peptide or be otherwise deleterious. So the pros and cons of taking this approach should be presented.
We have extended the end of this section as follows:
“Of note, there is no way to tell if the indel is a read-error or a synthesis error, thus the true amino-acid sequence of the bound peptide is not known. As a synthesis error may be translated to a deleterious change to the peptide sequence, BIPS allows identification with or without allowing for indels for that purpose, and we suggest users identify their needs accordingly.”

• Fig. 2 only referred to only in Supplementary/Methods?

Fig. 2a is referenced in the main text, but indeed Fig. 2b and Fig. 2c were only referenced in the methods section. This has been corrected.

• A typical peptide length and corresponding barcode length should be given with numbers. The qualitative descriptions are nice to read but some numbers to get an order of magnitude would be helpful.
We provided the default parameters within the Test Case section:
“We ran BIPS separately on each of these inputs, creating two separate libraries (with default parameters including oligo_aa_length=64, oligo_aa_overlap=20, and a 5-part barcode at the 5’ end of 15bp per part).”

• The sentence “Figures were created with Biorender.com” seems to be in the wrong place
Moved to figure 1 legend where it belongs.

• It seems in the text that the software has three options for barcoding, but after checking the code, it seems only be able to do the third. Please clarify.
Assuming the reviewer is referring to internal, external, and full sequencing identification, this is written directly when mentioning the three methods of sequence identification: “BIPS creates internal barcoding sequences by leveraging the multiple possibilities in coding amino-acids into oligos”.

• The example and default configurations should be mentioned in the methods text
Both example and default configurations are identical, and we have added the example configurations mentioning they are default in the Test Case section.

• hamming distance (2n+1) … please explain n for the unfamiliar reader.
We thank the reviewer for this note, as we were able to see an inconsistency between the two usages of *n* in our manuscript (in Main and in Methods). We changed *n* to be the number of errors to be able to correct, and explained its meaning in the Method section.

• Please clarify the meaning of this sentence: Due to computational limitations we performed each single simulation up to 10^7^ sequences
This sentence has been clarified:
“If there were no barcode construction failures, we halted each single simulation upon reaching 10^7^ sequences. ”

• “Multiple inserts” should be defined for the unfamiliar reader.

We have added the definition of “more than one oligo cloned into a single phage, by oligonucleotide restriction sites ligating to one another and creating 3, 5 or more oligonucleotide sequences in the same phage, the first in the original direction the second in reverse etc.”
